# ﻿Bug cemetery: a case study of terrestrial isopod distribution on a brick wall in the Czech Republic

**DOI:** 10.3897/zookeys.1101.76132

**Published:** 2022-05-18

**Authors:** Ivan Hadrián Tuf, Nelly Weissová

**Affiliations:** 1 Department of Ecology and Environmental Sciences, Palacký University, Olomouc, Czech Republic Palacký University Olomouc Czech Republic

**Keywords:** Isopoda, Oniscidea, synanthropic habitat, vertical distribution, woodlice

## Abstract

Although terrestrial isopods (Oniscidea) are primarily soil- and surface-dwelling invertebrates, they can also be found on tree trunks and walls. This study evaluated distribution patterns of terrestrial isopods on a brick wall during the first hours of night in autumn. Four species of terrestrial isopods were recorded with *Armadillidiumversicolor* being the dominant one. Terrestrial isopods were distributed from ground level up to a height of 2 m, but preferred a 70–80 cm height band. The highest number of active individuals was observed 3 h after astronomical dusk. Potential predators of terrestrial isopods were abundant during the same time and at the same height.

## ﻿Introduction

**Figure 1.**
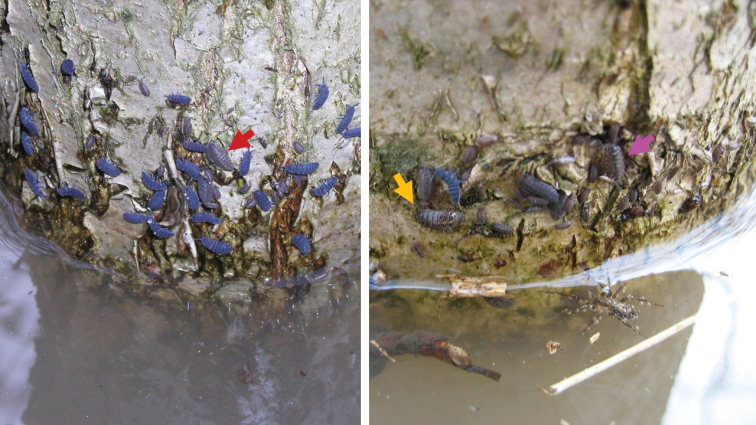
Springtails and terrestrial isopods climbing on tree trunks to avoid drowning during a spring flood. *Trachelipusrathkii* (Brandt, 1833), indicated by pink arrow, *Protracheoniscuspolitus* (C. L. Koch, 1841), indicated by orange arrow, and *Porcelliumconspersum* (C. L. Koch, 1841), indicated by red arrow, are visible. Litovelské Pomoraví PLA (Czech Republic), 30 March 2006 (photographs IHT).

Terrestrial isopods (Crustacea, Isopoda, Oniscidea) inhabit soil generally and feed on dead and rotting organic matter. They can be found in upper soil layers including the litter layer (Cole 1946). They shelter under bark, logs and stones or they are true troglobionts. There are several species climbing on vegetation as reported from tropical areas, such as Central America (e.g., Van Name 1936), Central (Schmidt 1999) and South Africa (Glazier and Kleynhans 2015) or St. Helena Island (Dutton and Pryce 2018). In Europe, there are only a few anecdotal reports about the presence of terrestrial isopods on trees, walls, and inside tree hollows for species of the genus *Armadillidium* (e.g., *A.pictum* Brandt, 1833, *A.depressum* Brandt, 1833, *A.pulchellum* (Zenker, 1798), *A.vulgare* (Latreille, 1804)), the genus *Porcellio* (*P.scaber* Latreille, 1804, *P.spinicornis* Say, 1818), *Oniscusasellus* Linnaeus, 1758, and occasionally other species (Růžička et al. 1991; Alexander 2008, 2011; Gregory 2009; Božanić et al. 2013; Tracz 2013; Boeraeve et al. 2021). This above-ground activity is sometimes related to heavy rains (Abbott 1918; Standen 1921; Cole 1946) or spring floods (Fig. 1). However, a recent systematic inventory of arthropods in oak canopies in Norway revealed five species of terrestrial isopods with high prevalence of *A.pictum* (Thunes et al. 2021). Sutton (1972) noted the presence of *P.scaber* on tree trunks and brick walls feeding on green algae.

Although the nocturnal presence of *P.scaber* on trees is commonly known (Sutton 1972; Cloudsley-Thompson 1977; Warburg 1993), there are only a few systematic studies. The first one was by Brereton (1957), who systematically searched for terrestrial isopods on tree trunks near Oxford for two years; he reported four species found on tree trunk bases but only two of them (*P.scaber* and *O.asellus*) also at eye-level height (~ 1.8 m). During the year, isopods were the most numerous on trees in spring and in September/October. Brereton (1957) confirmed that *P.scaber* overwinters in moss pillows at the base of the trunk and the species climbs on branches during late summer: he found them in high numbers at 5.5 m height in early September. High abundance of terrestrial isopods in moss on the base of tree trunks were caused by downward migration of isopods of the canopy and the trunk, as confirmed with the use of trunk traps (Brereton 1957). The surprising opposite pattern of seasonal migration on trees for overwintering of *P.scaber* was suggested by Fritsche (1934; Vandel 1962). Prevailing descent migration from trees during a year was revealed for *Spherillo* spp. and *Trichoniscus* spp. in New Zealand (Moeed and Meads 1983), indicating breeding in canopies.

Another exhaustive study of *P.scaber* living on trees near Den Haag, the Netherlands, was published by Den Boer (1961), who studied its activity in the same years as Brereton (1957), 1953–1954 and 1953–1955, respectively. He described parameters of daytime shelters on the bark for *P.scaber*. He found that very few isopods, active during the night on tree trunks with such shelters, travel down to hide in the litter layer during daytime. Another interesting finding (recorded using the capture-mark-recapture method) is that although isopods are present on the bark during the whole night, activity of each individual isopod spans only approximately one hour (Den Boer 1961).

Sixty years later, aspects of distribution of terrestrial isopods on tree trunks were presented at the 11^th^ International Symposium on Terrestrial Isopod Biology by Mechthold et al. (2021). They highlighted phenological differences between tree trunk active populations of *P.scaber* and *O.asellus* during the year.

Beside these studies on the activity of terrestrial isopods on tree trunks, there is only one paper studying distribution of isopods on walls. Cloudsley-Thompson (1958) counted terrestrial isopods on a stone wall at night and found that their numbers decrease with increasing wind speed. His explanation was that wind inhibits their activity due to a reduction of air humidity.

We also received anecdotal observations of terrestrial isopods climbing on walls of buildings during the night. In this study we present data on the distribution of terrestrial isopods on a brick wall in the Czech Republic to study (1) whether there is a temporal pattern in the distribution of particular species of terrestrial isopods, (2) whether its distribution depends on the air temperature or humidity, and (3) whether the distribution of predators corresponds with the distribution of terrestrial isopods.

## ﻿Materials and methods

**Figure 2.**
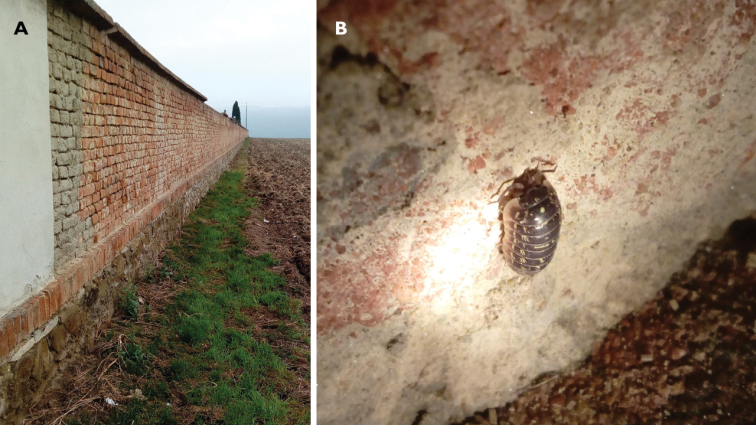
**A** the studied brick cemetery wall **B***Armadillidiumversicolor* climbing on the wall (photographs NW).

After a short pilot survey, we selected a study wall on which we found a high number of active terrestrial isopods during the night. This particular brick wall was found on the outskirts of the town of Kostelec na Hané (Czech Republic) at the local cemetery (49°31'06.0"N, 17°03'44.6"E). The length of the wall is 190 m and its height is ~ 2.5 m. The first 4 m of the wall is plastered, the rest are bare bricks standing on a 50 cm high stone foundation (Fig. 2A). Terrestrial isopods and their potential predators were studied along a 30 m long transect of the non-plastered part at the outward side of the wall (exposed to the east, i.e., influenced by the prevailing south-eastern wind) from the base to a height of 2 m. The wall was bordered by a freshly ploughed agricultural field and generally surrounded by an urban and agricultural landscape. The nearest forest is located at ~ 2 km. The nearest street lamp was located ~ 20 m away, but the studied side of the wall was not illuminated by artificial light.

Following Brereton (1957), the month of October (2019) was chosen as the best time to study terrestrial isopod activity. Previous observations indicated the highest abundance of terrestrial isopods between 21:00–23:00 h (i.e., 1.5–3.5 h after astronomical dusk which was at 19:38 on the first observation day; Fig. 2B). Therefore, terrestrial isopods and other invertebrates were observed daily from 20 to 26 October. Each daily observation started at ~ 21:15 (± 15 min) and lasted 90 min. At the start of each observation, we measured air humidity, air temperature, and light intensity (lux). For each observed individual, we noted species identity, time of observation, and height above the ground with a Dictaphone. Numbers of predatory invertebrates, i.e., centipedes, spiders, and harvestmen, were also noted to evaluate the pattern of distribution of potential predators. Observations were made using a hand torch with white light, since we were not interested in the behaviour of the animals, but only their position. No fleeing reaction was noted during the research. No individuals were collected during the observations. A few individuals for identification were taken a few days before the start of the first observation.

All data was entered into MS Excel, and we used CANOCO 5 (Šmilauer and Lepš 2014) for statistical analysis. Numbers of individuals of the different terrestrial isopod species were used as species variables. As environmental variables we used height (cm), air humidity (%), air temperature (°C), time after astronomical dusk (minutes), and numbers of individuals of predators (summed numbers of centipedes (Chilopoda), spiders (Araneae), and harvestmen (Opiliones)). We used a redundancy analysis (RDA) for visualizing the relationship between number of terrestrial isopods per species and environmental variables. We did not incorporate the effect of light intensity in the analysis, because measured values were very low and varied between 0 and 2 lx. Environmental variables that significantly explained variation in the terrestrial isopod distribution were used to calculate predictive GAM models (normal distribution of data).

## ﻿Results

**Figure 3.**
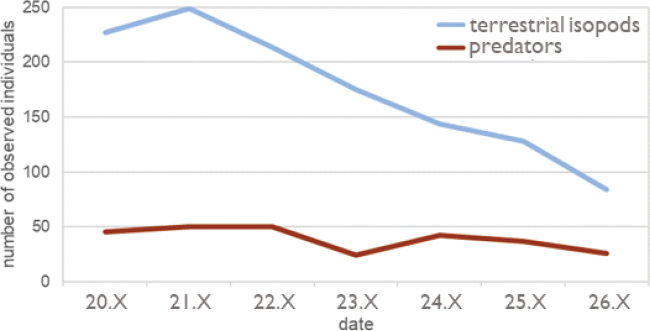
Number of invertebrates recorded per day on the wall during a 90-min observation during October 2019.

**Figure 4.**
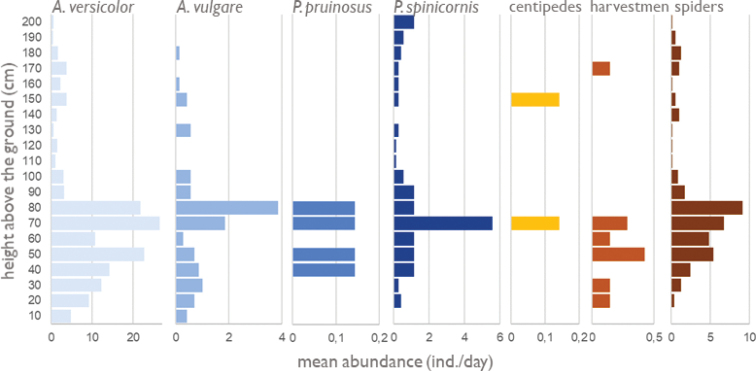
Vertical distribution of terrestrial isopod species and predators on the wall per day in 90-min observation.

In total, 1221 terrestrial isopods belonging to four species were observed. By far the most numerous was *Armadillidiumversicolor* Stein, 1859 (1020 individuals), followed by *Porcelliospinicornis* (112 ind.), *Armadillidiumvulgare* (85 ind.), and *Porcellionidespruinosus* (Brandt, 1833) (4 ind.). Altogether, 266 spiders and only two centipedes and nine harvestmen were observed. The number of observed terrestrial isopods and predators decreased during the sampling period (Fig. 3), which could be due to the decreasing recorded temperature. However, this correlation was not significant (Pearson’s r = 0.37 and p = 0.414 for temperature and number of observed terrestrial isopods, Pearson’s r = 0.48 and p = 0.276 for temperature and number of observed predators). The highest mean numbers of individuals for (almost) all species were recorded at a height of 70–80 cm (Fig. 4).

**Figure 5.**
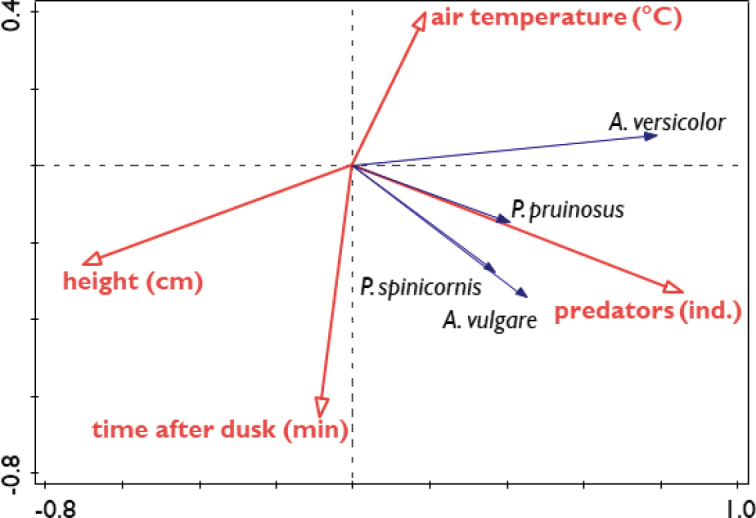
RDA-biplot for the distribution of different species of terrestrial isopods (blue arrows) on the wall during a 90-min observation in October 2019 and environmental variables (red arrows). Only environmental variables with a significant effect on terrestrial isopod distribution are presented.

**Figure 6.**
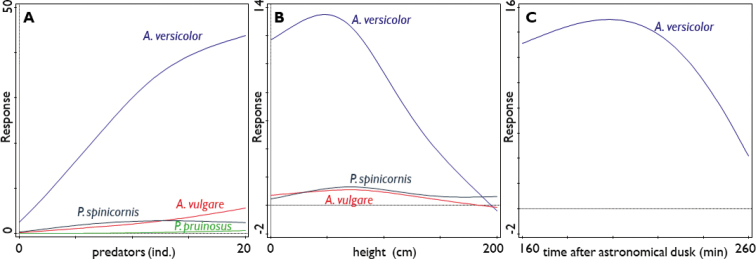
Significant response (see Table 1) of terrestrial isopod numbers per species to **A** number of predators **B** height on the wall **C** time after dusk during a 90 min interval in October 2019.

The RDA for predicting the distribution of terrestrial isopods using environmental variables (Fig. 5) was statistically significant (pseudo-F = 28.3, p = 0.002). The first and the second axes explained 47.2% and 3.6%, respectively of the variability in terrestrial isopod distribution. The strongest predicting variables were number of predators (explaining 35.2%, pseudo-F = 74.8, p = 0.002) and the height above the ground (explaining 12.2%, pseudo-F = 31.8, p = 0.002), whereas time after astronomical dusk and air temperature explained less than 2% of the variability in the terrestrial isopod distribution (1.8%, pseudo-F = 4.8, p = 0.002 and 1.9%, pseudo-F = 5.3, p = 0.01, respectively). The effect of air humidity was not significant (0.2%, pseudo-F = 0.8, p = 0.456). The presence of predators is a good predictor for the presence of terrestrial isopod species (Table 1, Fig. 6A), although *P.spinicornis* was most numerous at sites with medium numbers of predators.

**Table 1.** Summary of fitted Generalised Additive Models for environmental variables predicting the numbers of observed terrestrial isopods on the wall during a 90-min observation in October 2019. Significant effects in bold. (* p < 0.05, ** p < 0.01, *** p < 0.001, n.s. not significant).

Predictors	predators (ind.)			height (cm)			time after sunset (min)			air temperature (°C)		
Response	R2[%]	F	p	R2[%]	F	p	R2[%]	F	p	R2[%]	F	p
*A.versicolor*	**54.3**	**71.2**	***	**19.4**	**14.4**	***	**6.4**	**4.1**	*	4.3	2.7	n.s.
*A.vulgare*	**29.2**	**24.8**	***	**10.1**	**6.7**	**	4.5	2.9	n.s.	4.3	2.7	n.s.
*P.pruinosus*	**29.3**	**24.8**	***	3.4	2.1	n.s.	0.8	0.5	n.s.	3.1	1.9	n.s.
*P.spinicornis*	**23.4**	**18.3**	***	**10.3**	**6.9**	**	2.4	1.5	n.s.	0.7	0.4	n.s.

Based on GAM models, the number of terrestrial isopods was significantly predicted by the height of the record on the wall for all species except *P.pruinosus* (Table 1), the three other species were most numerous at a height of 70–80 cm above the ground (Fig. 6B). The time after astronomical dusk was a significant predictor for the number of observed individuals of *A.versicolor* (Table 1), which reached the highest numbers ~ 3 h after dusk (Fig. 6C). The air temperature had no significant effect on the prediction of the numbers of observed individuals of single terrestrial isopod species (Table 1).

## ﻿Discussion

We present data on the distribution of four species of terrestrial isopods on a wall at night in autumn 2019 in the Czech Republic. The distribution of the most numerous species was significantly explained by the height on the wall and for one species there was also a significant correlation between the number of observed individuals and the time after sunset. Distribution of all species had a significant relationship with the presence of predators, which mainly consisted of spiders.

All species of terrestrial isopods found on the studied wall are common inhabitants of cities in the Czech Republic (Orsavová and Tuf 2018). The absence of *P.scaber* is surprising because it is frequently found on walls of buildings in the nearby city of Olomouc (pers. obs.) and it was reported from walls several times (e.g., Den Boer 1961), including brick walls (Meinertz 1944). The only species found in this study and reported before from (limestone) walls is *P.spinicornis* (Sutton 1972; Boeraeve et al. 2021).

The preferred height of all species was ~ 70–80 cm, with low numbers of animals at 60 cm (Fig. 4). It is necessary to say that at a height of 60 cm there was a small prominent lintel between the lower stones and the upper brick parts of the wall (see Fig. 2A). For some reason, animals (both terrestrial isopods and spiders) were more numerous above and below this brick lintel. Higher numbers of observed individuals could be related to higher humidity near the lintel (catching rainfall) or small accumulation of debris including excrements (see Fig. 2B); probably without the presence of the lintel, distribution of terrestrial isopods on the wall would be less unequal.

The observed temporal pattern, with the highest number of observed individuals at approximately 21:30 h, is in accordance with the temporal activity pattern observed for *P.scaber* in the Oxford study (Brereton 1957). This species was active there from 19:00 till 03:00 h, with the highest observed activity around 23 h, indicating that its activity increased continuously the first hours after sunset. Brereton (1957) observed individuals of *P.scaber* mainly on well-structured bark of oaks and sycamores. On the other hand, Den Boer (1961) reported the highest numbers of *P.scaber* after sunset and before dawn. He supposed that individuals climb the tree trunks actively each evening from litter around the tree base because they are not able to find shelter on the smooth bark of studied aspens. The wall we studied is old with partly eroded ground between the bricks with many crevices offering a lot of shelter. We do not suppose that terrestrial isopods climb on the wall from shelters at or near the soil every night and therefore our data support the view of Brereton (1957) that the terrestrial isopods find daytime shelter on the wall.

We found a weak effect of air temperature and no effect of air humidity on the activity of terrestrial isopods. Cloudsley-Thompson (1958), as well as Den Boer (1961), reported an effect of air humidity in explaining the vertical activity of terrestrial isopods. However, we did not find this effect, which may be an artefact of the low variability in air humidity during our research (58–70%, one night of 46%), but it is also necessary to say that the genus *Armadillidium* is less vulnerable to low humidity levels than, e.g., *P.scaber* (Cloudsley-Thompson 1977) and has a higher drought resistance (Dias et al. 2013). On the other hand, Brereton (1957) did not find effects of air humidity, nor air temperature, on the activity of *P.scaber*.

The strongest predictive power for the numbers of observed terrestrial isopods on the wall was the number of observed predators. Centipedes, spiders, and harvestmen are known isopod predators (Cloudsley-Thompson 1958; Sutton 1970; Santos and Gnaspini 2002). The observed predatory individuals were the centipede *Lithobiusforficatus* (Linnaeus, 1758), the harvestman *Phalangiumopilio* Linnaeus, 1761, and the spiders *Nucteneaumbratica* (Clerck, 1757) and *Steatodagrossa* (C. L. Koch, 1838). Of course, terrestrial isopods do not prefer places with high predatory pressure, but the reason for the presence of predators at specific heights with high numbers of terrestrial isopods is probably due to accessibility of their prey or overall suitability of environmental conditions there. Nevertheless, we can conclude that high abundance of predators at a particular height could be a good predictor for terrestrial isopods on walls.

It is not yet clear exactly why terrestrial isopods are found on walls, but the search for algae as food source is most plausible. Brereton (1957) experimentally confirmed consumption of *Pleurococcus* algae from the tree bark by *P.scaber*. However, in the same experiment, algae were not consumed by *O.asellus* (Brereton 1957). We therefore encourage research that investigates feeding habits of terrestrial isopods on walls and on trees!

In conclusion, we observed four species of terrestrial isopods present on the wall during the several hours after sunset. They were distributed along the complete studied height (up to 2 m), but they preferred a height of ~ 0.75 m above the soil surface. Similar spatiotemporal patterns were recorded for spiders as their potential predators. We suppose that the terrestrial isopods shelter in fissures and crevices between bricks and that they are resident on the wall. It will be very useful to study their gut contents to discover what food is consumed, which will probably explain their distribution.
